# Secondhand smoke exposure at home and its association with tobacco use among adolescents in Saudi Arabia: findings from the 2022 global youth tobacco survey

**DOI:** 10.1186/s12889-026-28019-3

**Published:** 2026-06-08

**Authors:** Moroj A. Aldarmasi

**Affiliations:** 1 Preventive Medicine and Public Health Department, Faculty of Medicine, King Abdulaziz University, Jeddah, 21589 Saudi Arabia; 2 Preventive Medicine Unit, King Abdulaziz University Hospital, Jeddah, 21589 Saudi Arabia

**Keywords:** Secondhand smoke, Adolescents, Tobacco/nicotine product use, Saudi Arabia, GYTS, Logistic regression

## Abstract

**Background:**

Secondhand smoke (SHS) remains a preventable public health hazard that disproportionately affects adolescents. Despite the implementation of tobacco control measures in Saudi Arabia, SHS exposure within homes persists. This study assessed the prevalence of household SHS exposure among adolescents and examined its association with current tobacco/nicotine product use behaviors, including cigarettes, shisha, and e-cigarettes, using nationally representative data from the 2022 Global Youth Tobacco Survey (GYTS).

**Methods:**

A secondary analysis was conducted on the 2022 GYTS dataset for Saudi Arabia, comprising 6,983 students aged 11–17 years. Complex survey design weighting was applied to ensure national representativeness. SHS exposure was defined as at least one day of exposure to tobacco smoke at home in the past seven days. Weighted prevalence estimates were calculated, followed by Rao–Scott chi-square tests and multivariable logistic regression analyses adjusting for age, sex, parental and peer smoking, and weekly allowance.

**Results:**

Overall, 18.3% of adolescents reported SHS exposure at home. SHS exposure at home was higher among females (21.8%) than males (14.9%). Adolescents exposed to SHS at home had significantly higher odds of current cigarette use (AOR = 1.78; 95% CI 1.11–2.85), shisha use (AOR = 1.69; 95% CI 1.04–2.76), e-cigarette use (AOR = 1.74; 95% CI 1.15–2.63), and any tobacco/nicotine product use (AOR = 1.66; 95% CI 1.19–2.32) compared with their unexposed peers. Female sex, parental smoking, and having friends who smoked were independently associated with SHS exposure (all *p* < 0.05).

**Conclusions:**

Nearly one in five Saudi adolescents are exposed to secondhand smoke at home. Household SHS exposure was independently associated with higher odds of current tobacco/nicotine product use across multiple products. These findings underscore the need for family-centered interventions and strengthened efforts to promote smoke-free home environments to reduce youth tobacco vulnerability.

## Background

Tobacco use and exposure to secondhand smoke (SHS) remain among the leading preventable causes of morbidity and mortality worldwide [1, 2]. Despite substantial progress in tobacco control, involuntary exposure to SHS continues to affect millions of children and adolescents, both at home and in public places [3, 4]. Globally, exposure to SHS is responsible for more than one million deaths annually, with nearly half of these occurring among women and children [5, 6].

In Saudi Arabia, the burden of tobacco-related diseases remains high, and exposure of youth to tobacco smoke continues to be a significant public health concern [7, 8]. Although the country has ratified the WHO Framework Convention on Tobacco Control (FCTC) and enacted national tobacco-control programmes, data from recent surveys indicate that 12–20% of adolescents in Saudi Arabia still live in homes where others smoke and up to ~ 40% report exposure in public places, suggesting that smoke-free home environments are far from universally adopted [9, 10]. The Global Youth Tobacco Survey (GYTS) provides critical insights into the prevalence and determinants of tobacco use and SHS exposure among school-aged adolescents, serving as the principal national data source for monitoring progress toward FCTC Article 8 compliance [11].

Evidence from Gulf Cooperation Council (GCC) countries suggests that approximately one in four to one in three adolescents are exposed to SHS in household settings [9]. Studies conducted in Madinah and other Saudi settings have demonstrated strong associations between parental smoking and adolescent smoking behaviors [7]. However, limited research from Saudi Arabia and the broader Arabian Gulf region has examined the association between household SHS exposure and subsequent tobacco or nicotine product use among adolescents [6]. International evidence has shown that SHS exposure is associated with increased risks of nicotine dependence, respiratory illness, cardiovascular dysfunction, and initiation of active smoking among youth [12–14]. This association may be influenced by familial normalization of smoking behaviors, parental behavioral modeling, and reduced perception of tobacco-related harms among adolescents [15, 16].

However, few studies in the Arabian Peninsula have simultaneously assessed the prevalence of SHS exposure at home and its association with multiple forms of youth tobacco use—cigarettes, shisha, and e-cigarettes—using nationally representative data. To address this gap, we analyzed the 2022 GYTS data for Saudi Arabia to:

1. Estimate the prevalence of SHS exposure at home among adolescents,
2. Examine associations between household SHS exposure and current tobacco/nicotine product use behaviors,
3. Assess sex differences in household SHS exposure, and.
4. Identify sociodemographic and behavioral correlates of SHS exposure.

## Methods

### Study design and data source

This study employed a cross-sectional analytical design using data from the 2022 Global Youth Tobacco Survey (GYTS) conducted in Saudi Arabia. The GYTS is a nationally representative, school-based survey jointly coordinated by the World Health Organization (WHO) and the U.S. Centers for Disease Control and Prevention (CDC) [17, 18]. The survey utilized a two-stage cluster sampling design to collect information on tobacco use, SHS exposure, and related behaviors among adolescents aged 11–17 years.

Data collection was conducted during the 2022 academic year using anonymous, self-administered paper-based questionnaires completed during regular classroom sessions. Trained survey administrators supervised data collection following standardized GYTS protocols, while teachers were generally not involved during questionnaire administration to minimize response bias. Schools were selected proportionate to enrollment size across all administrative regions of Saudi Arabia and consisted of public intermediate schools. In the second sampling stage, classrooms within selected schools were randomly chosen, and all students in selected classes were eligible to participate.

A total of 6,983 students participated in the survey. Following data cleaning and exclusion of incomplete responses for specific variables, the analytical sample size varied slightly across regression models because of listwise deletion for missing data. The overall survey response rate was 85.9%, consistent with WHO standards for GYTS survey validity.

### Ethical approval

The study utilized secondary, de-identified data from the 2022 Global Youth Tobacco Survey (GYTS) conducted under the joint coordination of the World Health Organization (WHO) and the Saudi Ministry of Health. Ethical approval for the original survey was obtained from the Saudi Ministry of Health Institutional Review Board (IRB) in accordance with the Declaration of Helsinki. All participants provided informed assent, and parental consent was secured prior to data collection. Permission to use and analyze the dataset for this study was granted by the Unit of Biomedical Ethics at King Abdulaziz University Hospital (Reference No 504-25).

### Variables and measures

The primary exposure variable was SHS exposure at home, assessed using the question: “During the past seven days, on how many days has anyone smoked inside your home, in your presence?” Students reporting exposure on one or more days were categorized as exposed to SHS at home.

Primary outcome variables included current tobacco/nicotine product use during the past 30 days, including cigarette smoking, shisha smoking, e-cigarette use, heated tobacco product use, and any tobacco/nicotine product use, defined according to WHO GYTS guidelines [18]. Attitude-related variables included perceptions regarding the harmfulness of SHS from cigarettes and shisha, as well as support for smoke-free indoor and outdoor public places. These variables were assessed using standard GYTS questionnaire items and categorized according to participant responses.

Potential covariates included age (in years), biological sex, grade level, weekly allowance, parental smoking, and friends’ smoking status. Grade levels corresponded to the three grades of intermediate school education in Saudi Arabia. Weekly allowance was reported in Saudi Arabian Riyals (SAR), with categories approximately equivalent to: less than 30 SAR (<8 USD), 30–49 SAR (8–13 USD), 50–99 SAR (13–26 USD), and 100 SAR or more (≥27 USD). Parental smoking variables (father and mother smoking status) were included as indicators of the household smoking environment; however, the GYTS does not capture smoking by other household members such as siblings or extended family.

### Statistical analysis

All analyses accounted for the complex survey design using weighted estimates. Descriptive statistics were calculated for all variables. Weighted prevalence estimates with 95% confidence intervals (CIs) were computed using complex sample procedures. Associations between SHS exposure and current tobacco/nicotine product use were initially examined using Rao–Scott χ² tests to adjust for survey clustering.

Binary logistic regression analyses were then conducted to estimate crude and adjusted odds ratios (AORs) with 95% confidence intervals. Adjusted models included age, sex, weekly allowance, parental smoking, and friends’ smoking as covariates to control for potential confounding. Age was modeled as a continuous variable in the multivariable regression analyses. Parental smoking was treated as an independent covariate reflecting the household smoking context rather than as a mediator. Determinants of SHS exposure were examined separately using multivariable logistic regression. Multicollinearity among independent variables was assessed using variance inflation factors (VIF), with all VIF values below 2.5, indicating no evidence of problematic collinearity (Table 1). Model fit was evaluated using Nagelkerke’s pseudo R² and receiver operating characteristic (ROC) curve analysis. Model fit statistics for the regression models are summarized in Table 2. All covariates were entered simultaneously into the regression models based on theoretical relevance and prior literature rather than automated stepwise selection procedures.

**Table 1:** Multicollinearity diagnostics for predictors included in multivariable logistic regression analyses using variance inflation factor (VIF) statistics

Predictor	Tolerance *(SPSS Tolerance)*	VIF *(SPSS VIF)*
SHS exposure at home	0.838	1.193
Age	0.980	1.021
Sex	0.973	1.028
Weekly allowance	0.984	1.016
Father smoking	0.825	1.212
Mother smoking	0.894	1.118
Friends smoking	0.929	1.077

**Table 2:** Model fit statistics for multivariable logistic regression models predicting current tobacco/nicotine product use outcomes among Saudi adolescents

**Outcome**	**Model N ** ***(SPSS “Valid N”)***	**Nagelkerke R² ** ***(Pseudo R² table)***	**ROC-AUC ** ***(ROC output)***	**95% CI ** ***(ROC CI)***
Cigarette smoking	6267	0.175	0.739	0.720-0.757
Shisha smoking	6181	0.150	0.721	0.701-0.742
E-cigarette use	6421	0.188	0.732	0.713-0.751
Any tobacco/nicotine use	6591	0.142	0.676	0.662-0.690

*Abbreviations*:* ROC* receiver operating characteristic, *AUC* area under the curve

Missing data were minimal (<5% across all key variables) and were handled using listwise deletion within the complex samples framework. Sensitivity analyses comparing weighted estimates with complete-case analyses were conducted to assess the potential impact of missing data.

All statistical analyses were performed using IBM SPSS Statistics version 27.0 (IBM Corp., Armonk, NY), applying complex sample procedures. A two-sided *p*-value <0.05 was considered statistically significant. 

## Results

### Participant characteristics

A total of 6,983 adolescents aged 11 to 17 years were included in the analysis. As shown in Table 3, females comprised 49.0% of the weighted sample, and most were in the 13- to 15-year age range. Weekly allowance varied: about one-third reported spending less than 30 SAR per week, while 23.0% had no spending money.

**Table 3:** Weighted sociodemographic characteristics of participating adolescents aged 11–17 years in the 2022 Saudi Arabia GYTS

**Characteristic**	**Category**	**Weighted %**	**95% CI**
Age	≤11	1.9	(1.2-3.0)
	12	10.3	(8.7-12.3)
	13	27.7	(25.6-29.6)
	14	29.6	(27.9-31.6)
	15	22.9	(20.5-25.4)
	16	6.2	(5.0-7.6)
	≥17	1.5	(1.0-2.1)
Sex	Male	51.0	(45.5-56.2)
	Female	49.0	(43.8-54.5)
Grade	1st intermediate	35.1	(32.4-38.1)
	2nd intermediate	33.0	(30.1-35.1)
	3rd intermediate	31.9	(29.1-35.5)
Weekly allowance	None	23.0	(21.3-24.8)
	<30 SAR	35.4	(32.5-38.4)
	30–49 SAR	19.4	(17.8-21.0)
	50–99 SAR	12.9	(11.7-14.3)
	≥100 SAR	9.3	(8.0-10.8)

### Tobacco use and attitudes

Overall, 13.7% of respondents reported ever trying cigarette smoking, while 16.2% reported ever trying any tobacco or nicotine product (Table 4). Current (past-30-day) use of any tobacco/nicotine product was reported by 10.8% of adolescents. Current e-cigarette use (5.8%) exceeded both current shisha smoking (3.1%) and current conventional cigarette smoking (3.1%), while current heated tobacco product use was reported by 1.7% of participants.

**Table 4:** Weighted prevalence of tobacco/nicotine product use, SHS-related attitudes, and sensitivity analyses among Saudi adolescents

Variable	Response	Weighted %	95% CI	Complete-case prevalence (%)
Ever tried cigarette smoking	Yes	13.7	(12.1–15.5)	—
Current cigarette smoking (30-day)	Any days	3.1	(2.1–4.6)	2.5
Current shisha smoking (30-day)	Any days	3.1	(2.1–4.5)	2.5
Current smokeless tobacco use (30-day)	Any days	4.3	(3.3–5.9)	—
Current e-cigarette use (30-day)	Any days	5.8	(4.5–7.6)	4.1
Current heated tobacco product use (30-day)	Any days	1.7	(1.1–2.7)	—
Current tobacco/nicotine product use (30-day)	Yes	10.8	(9.5–12.3)	7.8
Believes SHS from cigarettes is definitely harmful	Yes	49.1	(46.4–51.8)	—
Believes SHS from shisha is definitely harmful	Yes	48.3	(45.7–51.0)	—
Supports smoke-free indoor public places	Yes	83.0	(81.3–84.7)	—
Supports smoke-free outdoor public places	Yes	74.9	(72.7–77.0)	—

Awareness of SHS-related harm was moderate, with 49.1% believing that cigarette smoke from others is definitely harmful and 48.3% reporting the same perception regarding shisha smoke. Support for smoke-free environments was high, with 83.0% supporting bans in indoor public places and 74.9% supporting bans in outdoor public places.

Sensitivity analyses comparing weighted estimates with complete-case prevalence estimates demonstrated no meaningful differences across tobacco/nicotine use outcomes, supporting the robustness of the findings (Table 4).

### Prevalence of SHS exposure

Overall, 18.3% (95% CI: 15.6–21.5) of adolescents reported SHS exposure at home during the past seven days. SHS exposure in indoor public places (27.0%; 95% CI: 23.2–31.4) and outdoor public places (28.1%; 95% CI: 24.4–32.3) was more prevalent, while 22.0% (95% CI: 19.9–24.3) reported exposure on school premises during the past 30 days.

Female students experienced significantly higher SHS exposure at home compared with male students (21.8% vs 14.9%, Rao–Scott χ² *p* < 0.001).

### Association between SHS exposure and current tobacco/nicotine product use

Bivariate analyses demonstrated consistently higher prevalence of current tobacco/nicotine product use among adolescents exposed to SHS at home compared with non-exposed adolescents (Table 5). Current cigarette smoking prevalence was higher among exposed adolescents than non-exposed adolescents (4.5% vs 2.2%, *p* = 0.018). Similarly, current shisha smoking (5.7% vs 2.8%, *p* = 0.012), current e-cigarette use (7.3% vs 3.6%, *p* = 0.009), and current use of any tobacco/nicotine product (13.8% vs 8.4%, *p* = 0.001) were significantly more common among adolescents exposed to SHS at home compared with non-exposed adolescents.

**Table 5:** Weighted prevalence and Rao–Scott adjusted chi-square test associations between SHS exposure at home and current tobacco/nicotine product use among Saudi adolescents

Outcome (30-day use)	SHS at Home Yes (%)	SHS at Home No (%)	Rao–Scott F (df1, df2)	*p*-value
Current cigarette smoking	4.5	2.2	6.26 (1, 30)	0.018
Current shisha smoking	5.7	2.8	7.15 (1, 30)	0.012
Current e-cigarette use	7.3	3.6	7.80 (1, 30)	0.009
Current tobacco/nicotine product use	13.8	8.4	13.29 (1, 30)	0.001

Heated tobacco product use was excluded from regression analyses because of low prevalence and unstable model estimates

Heated tobacco product use was not included in regression analyses because of its relatively low prevalence, which resulted in unstable model estimates.

### Multivariable logistic regression

After adjustment for sociodemographic and social factors, SHS exposure at home remained significantly associated with multiple forms of current tobacco/nicotine product use (Table 6). Adolescents exposed to SHS at home had 1.78-fold higher odds of current cigarette smoking (95% CI: 1.11–2.85), 1.69-fold higher odds of current shisha smoking (95% CI: 1.04–2.76), 1.74-fold higher odds of current e-cigarette use (95% CI: 1.15–2.63), and 1.66-fold higher odds of current use of any tobacco/nicotine product (95% CI: 1.19–2.32) compared with non-exposed adolescents.

**Table 6:** Multivariable logistic regression analyses examining the association between SHS exposure at home and current tobacco/nicotine product use among Saudi adolescents

Outcome	Crude OR	95% CI	Adjusted OR (AOR)	95% CI	*p*-value
Current cigarette smoking	2.12	1.30–3.46	1.78	1.11–2.85	0.017
Current shisha smoking	2.06	1.22–3.49	1.69	1.04–2.76	0.034
Current e-cigarette use	2.09	1.40–3.12	1.74	1.15–2.63	0.008
Current tobacco/nicotine product use	1.91	1.42–2.58	1.66	1.19–2.32	0.003

Adjusted for age, sex, weekly allowance, parental smoking, and friends’ smoking

### Determinants of SHS exposure

Predictors of SHS exposure at home are presented in Table 7. Male adolescents had significantly lower odds of SHS exposure at home compared with females (AOR = 0.60; 95% CI: 0.46–0.77; *p* < 0.001). Age was not significantly associated with SHS exposure after adjustment (AOR = 1.06; 95% CI: 0.97–1.16; *p* = 0.161).

**Table 7:** Multivariable logistic regression analysis of factors associated with SHS exposure at home among Saudi adolescents

Predictor	Adjusted OR (AOR)	95% CI	*p*-value
Male sex	0.60	0.46–0.77	<0.001
Age (years)	1.06	0.97–1.16	0.161
Father smokes	1.83	1.42–2.36	<0.001
Mother smokes	2.44	1.29–4.62	0.006
Friends smoke	1.71	1.29–2.28	<0.001

Reference categories were female sex, no parental smoking, and no friends who smoke

The model was adjusted for age, sex, parental smoking, friends’ smoking, and weekly allowance

Parental and peer smoking were significant predictors of SHS exposure. Adolescents with fathers who smoked had 1.83-fold higher odds of SHS exposure at home (95% CI: 1.42–2.36), while maternal smoking was associated with 2.44-fold higher odds (95% CI: 1.29–4.62). Having friends who smoked was also independently associated with increased odds of SHS exposure (AOR = 1.71; 95% CI: 1.29–2.28; all *p* < 0.001).

## Discussion

This nationally representative study provides updated evidence regarding SHS exposure among adolescents in Saudi Arabia using data from the 2022 Global Youth Tobacco Survey. Nearly one in five students reported exposure to SHS at home, and this exposure was significantly associated with current tobacco/nicotine product use across multiple products. These findings indicate that, despite national tobacco control policies, home environments remain settings where adolescents are frequently exposed to tobacco smoke.

The household exposure rate identified here (18.3%) is slightly lower than that reported in earlier GYTS waves conducted between 2010 and 2019 in Saudi Arabia and other Gulf Cooperation Council (GCC) countries, where adolescent exposure ranged from approximately 25% to 40% [7, 10] and which may reflect a gradual decline associated with broader regional tobacco control efforts [8]. This reduction may reflect the gradual implementation of national smoke-free legislation and improved awareness of indoor smoking bans [10]. However, the persistence of SHS exposure in home environments suggests that public-space smoke-free policies may not fully extend into private settings — a pattern documented in the Gulf Cooperation Council region [9] as well as in Jordan and other Arab states [19].

Our findings demonstrated that household SHS exposure was associated with significantly higher odds of multiple forms of current tobacco/nicotine product use among adolescents, including cigarettes, shisha, and e-cigarettes. Adolescents exposed to SHS at home had approximately 70–80% higher odds of current tobacco/nicotine product use compared with non-exposed adolescents. These findings are consistent with international evidence linking household smoking exposure with increased likelihood of tobacco initiation and continued nicotine use among adolescents [20]. This association aligns with broader evidence demonstrating the behavioral and physiological consequences of SHS exposure [12, 14].

Biological plausibility for these associations is well established. Exposure to SHS may contribute to nicotine sensitization, normalization of smoking behaviors within household environments, and reduced perception of tobacco-related harms among adolescents [16, 21, 22]. In the present study, these associations remained significant across conventional cigarettes, shisha, and e-cigarettes, suggesting that household smoking environments may influence perceptions and behaviors related to both traditional and emerging nicotine products.

It should be noted that the SHS exposure variable specifically measured exposure to combustible tobacco smoke within the home and did not assess exposure to e-cigarette aerosol. Therefore, the observed associations with e-cigarette use may reflect shared household norms, behavioral modeling, or normalization of nicotine products rather than direct exposure to aerosol. Future surveys may benefit from distinguishing between combustible smoke and electronic nicotine aerosol exposure in household settings.

### Sociodemographic and behavioral correlates

Female adolescents reported greater exposure to SHS at home, which may reflect sex-specific household patterns where women and girls spend more time indoors, while male smoking behaviors remain socially accepted [6, 7]. Parental smoking, particularly by fathers, was strongly associated with adolescent SHS exposure, consistent with findings from Saudi Arabia and neighboring Gulf states [7, 8, 10]. Having friends who smoke also increased exposure risk, reinforcing the social diffusion of tobacco use among youth networks. These findings emphasize the intertwined nature of familial and peer influences on adolescent tobacco behaviors. It is important to note that the GYTS item assessing SHS exposure captures smoking by any household member, whereas the available covariates assess only parental smoking. In multigenerational family structures common in the region, exposure may also arise from other relatives. Therefore, parental smoking variables in the present analysis should be interpreted as partial indicators of household smoking environment rather than definitive sources of SHS exposure. In the present analysis, parental smoking was modeled as a covariate to account for household smoking context rather than examined as a formal mediator, as the cross-sectional design limits causal pathway assessment.

### Public health implications

The persistence of SHS exposure highlights the importance of extending smoke-free initiatives into private spaces through culturally sensitive, family-based interventions. Health promotion initiatives should target parents as key agents of behavioral change and emphasize the harms of SHS exposure to children. Community health centers and school health programs can serve as strategic venues for awareness initiatives and cessation support for parents. Moreover, integrating tobacco prevention and smoke-free home education initiatives within Saudi Vision 2030 national health objectives may contribute to further reductions in youth tobacco exposure and nicotine product use [23].

### Strengths and limitations

Strengths of this study include its use of a large, nationally representative dataset and standardized GYTS methodology, enabling international comparability. However, several limitations warrant mention. The cross-sectional design precludes causal inference, and SHS exposure was self-reported, which may introduce recall bias. Due to the cross-sectional design of the GYTS, temporal relationships between household SHS exposure and tobacco use cannot be established. Therefore, causality cannot be inferred, and the observed associations should be interpreted as correlational rather than causal. Longitudinal studies are needed to clarify directionality. In addition, the survey did not capture household ventilation practices or enforcement of home smoking bans. Furthermore, although SHS exposure was defined as smoking by anyone inside the home, the survey measured only parental smoking status and did not assess tobacco use by other household members. In multigenerational households, this may result in unmeasured sources of exposure and residual confounding. Despite these limitations, the study provides important nationally representative evidence regarding SHS exposure and its association with current tobacco/nicotine product use among adolescents in Saudi Arabia.

## Conclusions

SHS exposure at home remains a significant public health concern among adolescents in Saudi Arabia. Household exposure was independently associated with higher odds of current cigarette smoking, shisha smoking, e-cigarette use, and overall tobacco/nicotine product use.

These findings underscore the importance of strengthening family-centered smoke-free initiatives, expanding parental smoking cessation support, and reinforcing national tobacco prevention strategies aimed at reducing adolescent exposure to SHS and nicotine products.

## Data Availability

The dataset analyzed in this study is available through the World Health Organization (WHO) GYTS data repository.

## Abbreviations

SHS: Secondhand smoke
GYTS: Global Youth Tobacco Survey
FCTC: Framework Convention on Tobacco Control
GCC: Gulf Cooperation Council
WHO: World Health Organization
AOR: Adjusted Odds Ratio
CI: Confidence intervals
CDC: Centers for Disease Control and Prevention
ROC: Receiver operating characteristic
VIF: Variance inflation factor
